# Protein Misfolding and Aggregation in Proteinopathies: Causes, Mechanism and Cellular Response

**DOI:** 10.3390/diseases11010030

**Published:** 2023-02-09

**Authors:** Mohammad Rehan Ajmal

**Affiliations:** Physical Biochemistry Research Laboratory, Biochemistry Department, Faculty of Science, University of Tabuk, Tabuk 71491, Saudi Arabia; mkhan@ut.edu.sa or ajmal.rehan@rediffmail.com

**Keywords:** protein aggregation, amyloid diseases, aggregation inhibition, inflammation, neurodegeneration

## Abstract

Proteins are central to life functions. Alterations in the structure of proteins are reflected in their function. Misfolded proteins and their aggregates present a significant risk to the cell. Cells have a diverse but integrated network of protection mechanisms. Streams of misfolded proteins that cells are continuously exposed to must be continually monitored by an elaborated network of molecular chaperones and protein degradation factors to control and contain protein misfolding problems. Aggregation inhibition properties of small molecules such as polyphenols are important as they possess other beneficial properties such as antioxidative, anti-inflammatory, and pro-autophagic properties and help neuroprotection. A candidate with such desired features is important for any possible treatment development for protein aggregation diseases. There is a need to study the protein misfolding phenomenon so that we can treat some of the worst kinds of human ailments related to protein misfolding and aggregation.

## 1. Introduction

**Figure 1:**
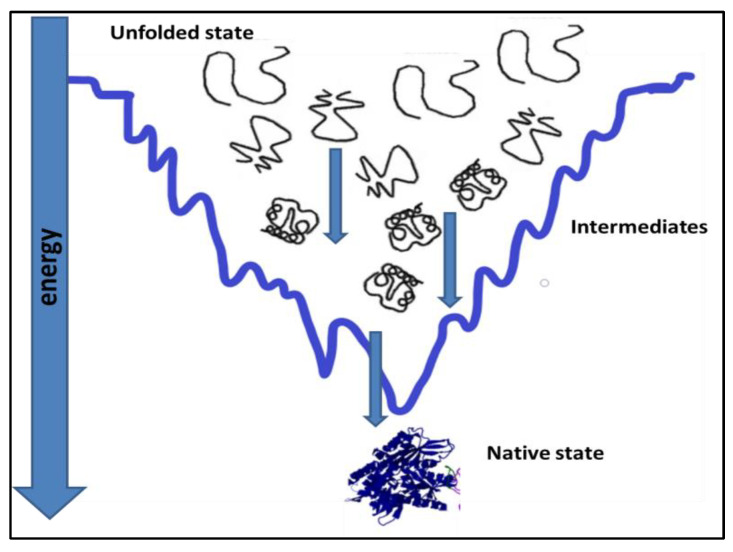
Attainment of native state conformation for a protein by a folding pathway [7,8].

Proteins, under physiological conditions, tend to remain most stable [1,2]. To achieve stability, the polypeptide chain folds itself in a way to attain the native state, which is the most stable state under prevailing physiological conditions, Figure 1. Protein folding in the cell is affected by its primary sequence and cellular environment [3]. Cells offer elaborate succor to folding by ATP-dependent chaperones [4]. Most of the time, protein folding is successful, and fully functional native protein structures are generated at the end of protein folding [5,6]. However, unsuccessful protein folding results in misfolded proteins. Many diseases are caused by misfolded proteins. Misfolding is an important reason for the loss of function in the misfolded state of the protein [7,8,9].

**Figure 2:**
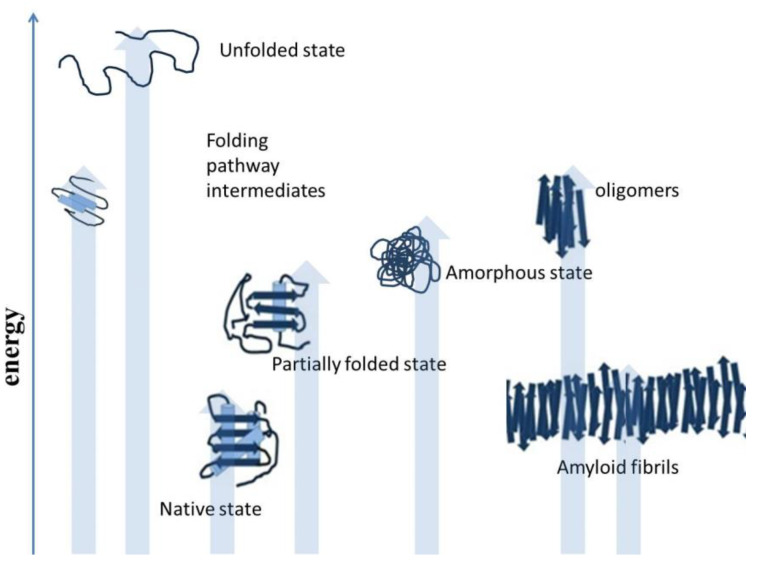
Deviation from the native structure leads to the formation of misfolded intermediates and aggregates [18].

Misfolding results when polypeptides deviate from the correct protein folding pathway [9,10], and misfolding occurs both inside and outside of the cells. Protein misfolding is accompanied by diverse structural morphology of polypeptides, as shown in Figure 2. The accumulation of misfolded protein aggregates as inclusion bodies, in the case of industrial protein production processes, constitutes a major concern. In the human body, aggregated proteins are linked to the pathology of many diseases. Misfolded polypeptides nonspecifically associate together and form aggregates [11]. Although, there are reports that protein aggregation involves specific interactions. Some protein motifs are more prone to aggregate formation. However, for in vivo protein deposition, the role of specific interactions is still unclear. Studies have reported the applicability of various experimental approaches to predict the amyloidogenicity of proteins [12]. There are self-aggregating proteins. These proteins are highly prone to aggregation, such as foot-and-mouth disease virus capsid protein and amyloid beta peptide [13]. There are many studies that showed similarity in the ultrastructure of intracellular aggregates and extracellular aggregates. Oligomers can play an important role in the organization and assembly of amyloid fibrils and intracellular protein inclusions [14]. Selective interactions motivate protein aggregation. Both oligomeric and fibrillar structures displaying amyloid-like properties are involved in the aggregation process. Self-aggregating oligomers of proteins can act as effective seeds in the formation of amyloid fibrils of the protein [15]. There can be conserved mechanisms to cause protein aggregation in different organisms, such as prokaryotes and eukaryotes. The studies focused on protein aggregation are important for the production of recombinant polypeptides for biotechnological and biomedical applications. There are specific circumstances which can direct cells to certain reorganizations and adjustments in the protein folding process. Proteins in the form of intracellular bodies can serve as adaptive mechanism to re-organize cellular functions in response to certain conditions. Protein inclusion in cells comprises proteins or protein-mRNA complexes that have functional roles in cells. These protein-mRNA complexes control cellular processes by restraining enzymatic activities, affecting gene expression, and mitigating DNA damage [16,17]. Protein-protein association is important to cells, such as host-pathogen interactions and stress responses mediated by various proteins, which play an important role in life. Some other functions of protein-protein associations include protein quality control and the replication process. Protein interaction events are controlled and are guided by chaperone proteins to minimize unwanted protein coalescence events.

Recent years have witnessed new findings regarding the handling of aggregated protein by cells. The cellular response in this event is highly regulated even if there is a coordinated coalescence of specific proteins forming intracellular bodies. Intracellular protein bodies vary in their physicochemical characteristics [19]. Physically they can exist as liquid, amorphous, and amyloid states. They have variable stability and turnover rates. Most physiological protein inclusions are reversible structures. Generally, after the inducing signal is removed, these intracellular inclusions do not leave any traces of their existence. Upon nutrient depletion, reversible enzyme deposits have been detected in cells [19]. While some proteins, such as prions, are signal-induced, self-maintaining structures and irreversible. Prions are abnormal proteinaceous pathogenic agents. They are transmissible and can induce abnormal folding of specific normal cellular proteins. Prions are found most abundantly in the brain. Prions are misfolded, and they have spatial properties to induce changes in the shape of similar proteins. Prions are known to cause amyloids in the brain and have associated brain disorders. Many times, protein bodies are not confined to individual cells and thus spread to the progeny cells during cell division [20,21,22]. Protein assembly is important for cells and performs critical functions, but for most coalescence bodies, relatively little is known. The assembly and disassembly of these bodies is regulated [23]. Changes in the chemical environment, such as pH, can affect their formation. Chemical modification, such as phosphorylation status, can affect protein body formation. In addition, there are enzymes such as aggregases that promote the assembly of coalescence bodies. The mechanism of inclusion into body formation assisted by aggregase is not fully understood. Further inquiry is needed to know substrate identification and singular deposit formation by aggregases [24,25]. Similar to their formation, disaggregation of coalescence bodies is also enzyme mediated or sometimes independent. In yeast cells, disaggregase Hsp104 plays an important role as disaggregation machinery. Hsp104 is necessary for the propagation of all of the amyloid-based yeast prions [26,27]. Hsp104 is absent in multicellular eukaryotic organisms. In metazoans, disaggregation machinery is comprised of Hsp40, Hsp70, Hsp110, and other small heat shock proteins [28,29]. In yeast, there is an efficient system of Hsp104 that helps in clearing even amyloid aggregates [30]. There are heritable prion states in yeast. These states basically necessitate Hsp104 disaggregation activity to produce amyloid seeds called propagons. In metazoans, these propagons are capable of dispersion into a new population in post-mitotic cells. In Alzheimer’s disease, hyperphosphorylated tau and β-amyloid (Aβ) are accumulated. This disease currently lacks effective treatment. Chaperone proteins are important targets. Heat shock protein (Hsp) 90 forms macromolecular complexes with co-chaperones. Hsp 90 complexes can regulate tau metabolism and Aβ processing [27,30,31]. Small molecule inhibitors of Hsp90 have been successful at ameliorating tau and Aβ burden. Pharmacology considerations of current scaffolds are hampering the development of these molecules as drugs. Other approaches are being considered to improve these compounds and to target co-chaperones of Hsp90 in an effort to limit these issues. Perhaps multicellular eukaryotic organisms are known to have functional amyloids [32]. Resistance and co-evolution of the physical state of aggregates may have occurred with disaggregation machinery. Disassembly machineries or diverse aggregation states and coalescence bodies need coordination for successful survival. Disaggregation is many times a substrate-tailored process based on the biological significance of aggregation state. There exists a fine balance between protein coalescence and sorting elements for their substrates, which is vulnerable to trepidations caused by abnormal protein aggregation. Abnormal protein aggregation may occur due to misfolding, which may be due to natural error or induced. Experimental studies on the aggregation process of stress-induced proteins have been conducted to explore this area. Stress-induced over-expression experiments revealed information about phase transitions in endogenous proteins. These studies showed that phase changes under physiological conditions affect the assembly of proteins, and this process can lead to perturbations of functional protein bodies and can result in pathological protein aggregation. Protein aggregation affects cellular functions. It is important to explore the large variety of adaptive reactions triggered by protein coalescence. In cells, the misfolded polypeptides are subjected to sorting by cellular proteasome machinery [33,34]. Degradation of misfolded protein ensures the accuracy of protein folding outcomes, which is important for normal cellular functions [35,36,37]. Protein misfolding diseases can relate to sequence alterations due to genetic defects, such as in cystic fibrosis. Cystic fibrosis is a progressive disease and is inherited. This disease affects the lungs and other vital organs [38]. Cystic fibrosis diseases arise as a result of mutations. In cystic fibrosis, the trans-membrane conduction regulator gene is affected, and a faulty gene result in abnormal protein function in the lungs and other parts of the body [39]. Other diseases arising from protein misfolding include prion diseases, Parkinson’s disease, and Alzheimer’s disease [40]. The hallmark of these diseases is aggregates and amyloids. In prion diseases, sporadic or acquired diseases are more common. A small percentage is linked to gene mutations. In humans, the PRNP gene forms prion proteins. Although the precise function is still unclear, researchers have suggested the involvement of prion proteins in the transport of copper into cells, assistance in neural communication, and neuroprotective functions. The prion gene mutation is linked to many familial prion diseases, such as Creutzfeldt-Jakob disease (CJD), Gerstmann-Sträussler-Scheinker syndrome (GSS), and fatal familial insomnia (FFI). Aggregate formation in proteins includes conformational alterations leading to aggregation of proteins in the cells and outside the cells. When aggregation occurs outside cells, it is not in the control of intracellular quality control systems. Accumulating misfolded and aggregated proteins is toxic to cells leading to injury and impairment of cellular function [41,42]. Proteins, when aggregated, possess altered physical properties that can be a major cause of malfunction in aggregated and misfolded proteins [43,44,45]. Excess glutamine repeats in mutated Huntington protein results in abnormal expansion of a glutamine stretch. After this change the protein is more susceptible to aggregate formation. Toxic accumulation of aggregated protein can occur in cells leading to impaired function [46,47]. Amyloids are protein aggregates with a fibrillar structure typically fibrils are 7–13 nm in diameter. Amyloids have predominantly beta sheet secondary structure and ability to be stained by dyes such as Congo red.

## 2. Protein Misfolding, Protein Aggregation and Amyloid Formation

**Figure 3:**
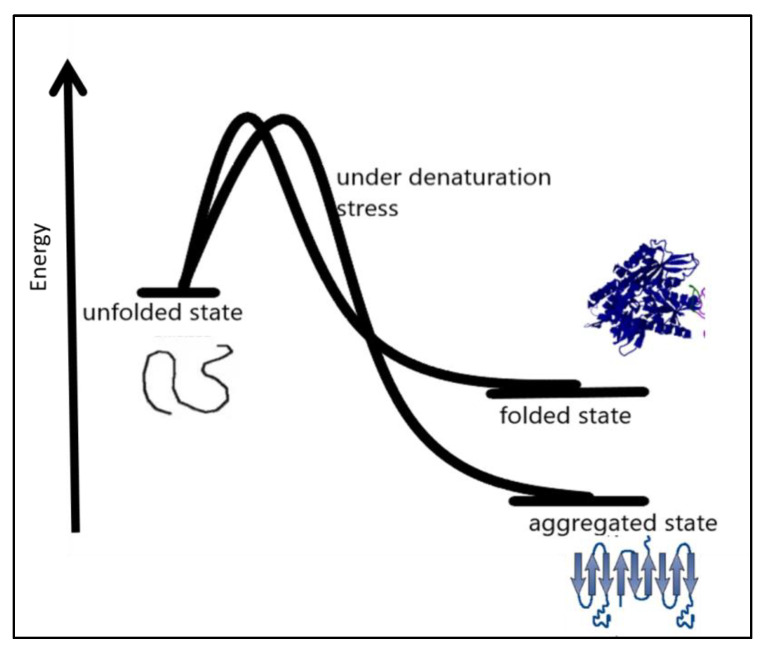
Under certain denaturation conditions, a protein moving into an aggregated state is more energetically favorable than the native state [55].

In many amyloid diseases, toxic functions have been attributed to protein aggregation in the extracellular space [48]. Precise coordination of the process of protein biogenesis, traffic, and homeostasis spatially in the early secretory compartment is very important for the maintenance of normal protein function. Disturbances in these processes may result in diseases [44,49]. Therapeutic approaches to this protein folding problem include molecules targeting different aspects that favor protein misfolding and aggregation [36,50]. Therapeutic molecules can be used to enhance the stability of the native conformation of proteins widening the thermodynamic barrier to protein misfolding and miss-assembly [51,52,53,54]. Another approach can focus on the clearance of preformed misassembled and misfolded aggregates of the proteins. Under certain conditions such as denaturation conditions, it becomes thermodynamically favorable for a protein to move into an aggregated state more stable than the native state under prevailing conditions, as shown in Figure 3.

Factors affecting protein folding include errors in post-translational modifications, protein environment alterations [56], increased degradation and accumulation of degradation products, and oxidative stress. Aggregation-prone proteins show increased aggregation at high protein concentrations [57]. Partially folded or misfolded intermediates have patches of surface hydrophobicity which make assembly a more easy and energetically feasible process, as shown in Figure 3. This assembly grows to oligomers, protofibrils, and fibrils of aggregated protein [58]. The amyloid fibrils are essentially an unbranched bundle of 2–6 filaments twisted together. These fibrils can interfere with biological functions and have been implicated in many neurodegenerative and cognition diseases [59]. Recently aggregated oligomers have also been linked to the pathogenicity of protein aggregation. Protein misfolding occurs when molecules are trapped in local energy minima with non-native architecture and properties [60]. The native structure is the most stable folded form for a protein under normal conditions. The folding pathway can be represented as an energy funnel with minimum energy and almost fixed conformation compared to other states. The native state of protein lies at the bottom of the funnel [61,62,63]. The energy of the intermediates decrease as they gain more ordered structure and approach the native state. The native structure generally has a packed structure with secondary elements, and tertiary structure. The achievement of native structure type depends on sequence and length of polypeptide. As the folding proceeds the number of molecules in different structural states decreases as most of them attain a native-like state, and finally, they all conclude in the native state. The native state is the most stable minimum energy state under a set of conditions. Small proteins fold by nucleation condensation mechanisms where small regions fold first, and then it guides the folding of the rest of the structure. In such cases, the nucleus of condensation or the region of the protein that guides the rest of the polypeptide to fold is important. If by any means this nucleation is affected, it can result in misfolding and aggregation of protein [64,65]. Protein misfolding and aggregation are linked to numerous human diseases and the aggregation of therapeutic proteins produced by recombinant techniques. Bioanalytical techniques based on optical readout of aggregation process and fibril formation have been developed. Recent times have witnessed tremendous growth in analytical method development for studying equilibrium between soluble and insoluble protein and aggregates within living systems. The field of protein aggregation and inclusion body formation has witnessed numerous conceptual turns. Natural and functional amyloid has emerged as separate domains of the protein aggregation phenomenon. There is immense possibility of exploiting amyloids for beneficial purposes such as natural biomaterials generation materials for various possible applications in industry and medicine. Some ambitious and highly desired recent research shows possible application of amyloids in water filtration technology [66,67,68]. Amyloids showed promising application possibilities in water purification. Including the removal of micro pollutants from water resources to produce potable water to quench the thirst of millions. To fully exploit aggregation, it is equally important to understand the process of aggregation itself. The creation of deviant interactions is caused by misfolding and mis-binding and starts the process of protein aggregation. These events expose beforehand hidden regions in polypeptides establishing unwanted contacts. These interactions result in self-assembly and the formation of large and insoluble aggregates.

Morphologically, amyloids exist as amorphous aggregates and fibrils [69,70,71]. Amorphous aggregates have a granular appearance in electron microscopy and are formed mainly by disordered polypeptide chains [72,73].

Amyloid fibrils are ordered and have repetitive structure elements. Such peculiar morphology is important in the context of the pathogenic and functional roles of amyloids. An important protein in the context of human diseases is the prion protein, and there is a strong link between misfolding and aggregation of this human protein in pathogenesis. The prion is an anchored helical protein in the cell membrane. The functions of prion protein include modulation of signal pathways, defense against oxidative stress, embryonic cell adhesion, copper binding, and maintenance of peripheral myelin.

Functional protein aggregates and functional amyloids have been discovered in various organisms. Prokaryotes, such as bacteria, and modern animals, including humans, have functional amyloids. During the evolutionary course, functional amyloids are optimized to self-assemble. They are conformationally more stable and resistant to damage than pathological amyloids. In addition, their assembly is faster. Functional amyloids are kinetically and thermodynamically favored to form ordered aggregated structures, impeding the buildup of highly harmful small metastable aggregates before reaching the final aggregated state [74].

Neurodegenerative conditions currently lack effective pharmaceutical treatment. Identifying and applying compounds that can target the multistep protein aggregation process is complicated and difficult. Therapeutics capable of perturbing the protein aggregation process are sought-after drugs [75]. Pathological protein aggregation of peptides and proteins can cause molecular and cellular pathogenicity and hence need attention by researchers to generate possible treatment plans for curing and treating these conditions.

There are reports of the aggregation of numerous peptides and proteins involved in important diseases. This problem is aggravated when it comes to aggregation-prone proteins. Such aggregation-prone proteins have been linked to the onset of many neurodegenerative diseases causing increased burden to caregivers and decreased quality of life for patients. Alzheimer’s disease, Parkinson’s disease, and Type 2 diabetes are some widely discussed and known names in this category [76,77,78]. Various amyloidogenic proteins can be trimmed to as few as five residue self-recognition elements. These self-recognition element retains the ability to aggregate and can serve as a starting point for aggregation. These self-recognition elements are important targets for aggregation inhibitors, as recent studies point to their significance in amyloid generation [15,79].

**Figure 4:**
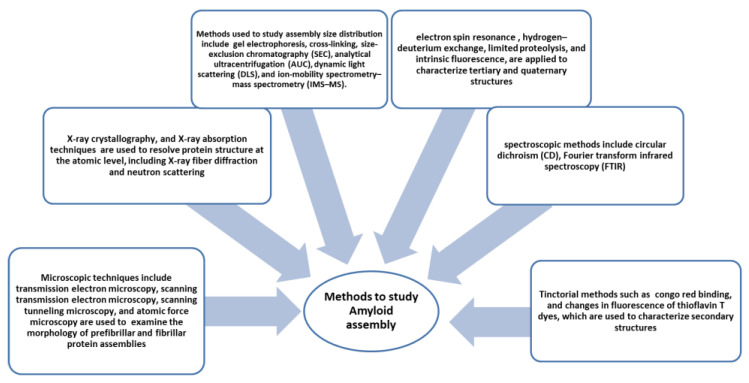
Methods and instrumentation applied to study amyloid formation [84].

Amyloid formation is a peculiar feature noticed in all amyloid diseases. Over 30 diseases have been reported to be caused by the deposition of insoluble aggregates. Analysis shows amyloid deposits bind congo red and have cross-β X-ray diffraction patterns [80]. Electron microscopy shows the morphology of amyloids as straight, unbranched fibrillar structures. There are various methods and techniques developed to study amyloid structure; some of them are shown in Figure 4. Under suitable inducing conditions, any protein can form amyloid. It has been suggested that aggregation is a primordial event, a highly stable polypeptide form that is intentionally and resourcefully overcome to create globular functional proteins. It is very interesting to know that non-disease fibrils formed by proteins also do not show cellular toxicity [81,82,83]. Thus it is imperative to study the structures and assembly of amyloids. Mechanisms of globular protein conversion to amyloid precursors and amyloid fibril structures need to be elucidated to understand the molecular events resulting in the pathogenicity of amyloids.

## 3. Cellular Response to Protein Misfolding and Aggregation

**Figure 5:**
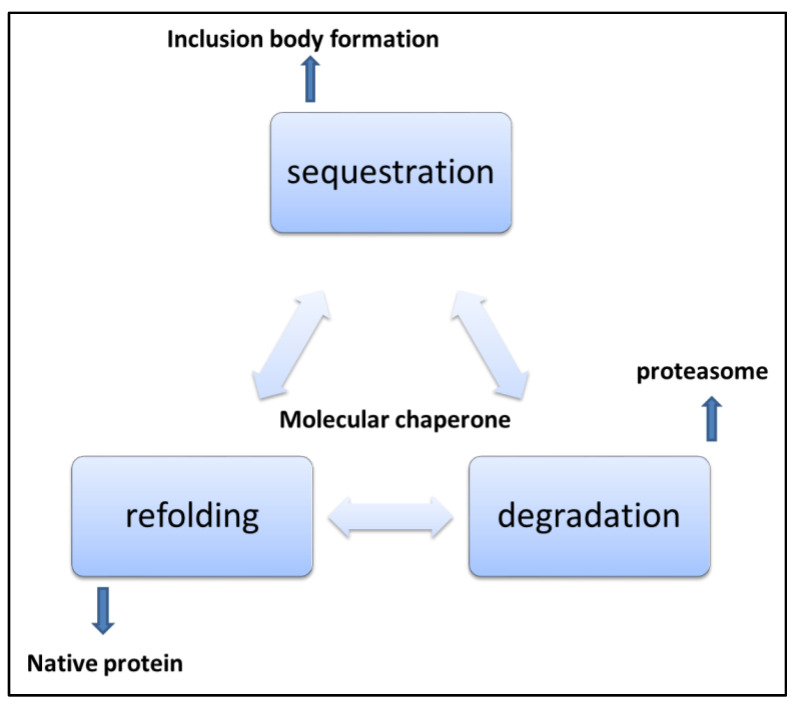
Mechanisms of handling misfolded and aggregated proteins [101].

The cellular protein quality check system continuously works, although its efficiency depends on many factors. Cellular, bodily, and environmental factors can affect the protein degradation system [85]. Protein homeostasis and proteostasis are two important interlinked phenomena in the protein quality control of cells [86]. The cellular environment is complex and crowded. The cellular mechanism to facilitate correct folding and removal of misfolded proteins is important [87,88]. Cellular protein quality control mechanisms aim to reinforce proper protein folding, prevent folding peptides trapped in misfolded structures, and remove and clean up already misfolded peptides from the system. Protein quality control sorts out proteins that have an increased possibility of aggregation and include mostly misfolded proteins. Cells have to spend energy for this process. Most of the time protein quality systems are ATP consuming processes for the cell. In eukaryotic cell there is compartmentalisation and protein folding is organelle specific. Different micro environments in different cell compartments result in a variety of challenges to protein folding, and thus different issues related to folding in organelle-specific chemical milieu exist [89]. Protein quality control in mitochondria is complicated as about 1500 proteins are in the mitochondria, most of which are imported from the cytosol. Only a few, less than 20, are encoded by the mitochondrial genome [90,91]. Mitochondrial proteins imported from cytosol are mostly post-translationally modified and move into mitochondria via the membrane pores in the inner and outer mitochondrial membrane [92]. Mostly proteins are imported in an unfolded state. This protein transport generally occurs at adhesion sites of the outer and inner mitochondrial membranes, and the transport process is governed by chemical gradient or ATP hydrolysis [93]. Similar mechanisms operate in the transport of nucleus-derived proteins to chloroplasts in plants. This process is generally ATP or GTP driven. N-terminal signal sequences in such peptides help them to be translocated to specific organelles. In some proteins, there also exists an internal signal sequence which is revealed when the terminal sequence is removed [94]. This internal signal sequence also helps in translocation. Mitochondrial chaperone HSP 70 binds incoming peptides and helps them pull into the organelle. Mitochondrial stress can develop because of accumulated and misfolded proteins, and damaged translocated proteins. Dysregulation in the translocation of nuclear-encoded mitochondrial proteins, disturbances in gene regulation of mitochondrial expressed proteins, and damage to mitochondrial DNA or its localized proteins can serve as a reason for mitochondrial stress. The endoplasmic reticulum is also an important site in cells where proteins are synthesized, and they attain specific functions after post-translational modification. The ER also plays an important role in protein sorting in the cell [95]. The ER lumen is a hub of molecular machines. Most importantly, various chaperones help the protein to gain its biologically active form while the protein gains post-translation modifications to prepare to perform some of the critical functions in the cell. In the ER, proteins are folded, co- and post-transnationally modified, and sorted for delivery into different cell compartments and cell locations. The ER has a specialized chaperone system assisting with the processing and post-translational protein achievement into the active state [96]. Intra luminal ER calcium reserves are important for proper ER functions. Perturbations in ER homeostasis impact protein misfolding and accumulation in the ER. Several ER chaperones need optimum calcium levels to retain their protein folding activity. The ER is a protein-folding factory for secretory pathways [97]. The ER hosts protein quality control mechanisms that are unique to it, important protein modifications such as glycosylation, disulphide formation, and other specialised modifications occur here. Quality control, in the ER protects cells from the accumulation of aberrant proteins and a specialised type of endoplasmic reticulum fusion system operates with lysosome for lysosomal destruction of the part of ER containing aggregated proteins [98]. Additional mechanism operates to check on the fidelity of post translational processes and survey proteins at early stages of synthesis and modification. Generally, the physical state of proteins such as hydrophobic patch exposure, glycan immature trimming, content of unpaired cysteine, and disulphide bond formation affects the tendency of proteins toward aggregation and are important recognition elements in ER protein quality control. Proteins interact with ER quality control components and are targeted for elimination or expulsion to cytosol where they are degraded by cytosolic proteasome machinery. Cell viability depends on proteome integrity. Changes in cells during ageing, cancer, stress conditions, unique metabolic changes, mutations and other stochastic events can affect the ER protein quality system. [99]. Protein misfolding and aggregation has emerged as a major mechanism for human diseases. The protein conformational disease list is growing day by day. With aging and other factors, cell’s ability to deal with the proteome decreases and is a major cause of late-onset diseases. Cytosolic protein quality components regularly search for possible substrates by binding to them in equilibrium of assembly and disassembly to prevent nascent proteins from misfolding and aggregation, as shown in Figure 5. Both nascent and pre-existing proteins are exposed to the aggregation process in the cytosol [100]. These events of protein aggregation can perturb cellular functioning and can enhance aging. Irreparable proteins are identified and subjected to elimination by quality control mechanisms. Mutations and adverse physiological conditions can lead to protein disorders due to protein aggregation. The difficulty for a protein to attain its native state can cause misfolding. Small molecules binding specifically to the folded state of a protein and stabilizing the structure are important candidates for the development of therapeutics. As protein aggregation prevents protein molecules from folding properly, these small molecules bind specifically to proteins or their intermediate folding states. They help stabilize the structure and are a good hope for the treatment of spatially misfolding-prone proteins related to a variety of human maladies.

In eukaryotes, the ubiquitin-proteasome system and macroautophagy are important protein cleanup mechanisms. Proteins destined for destruction are targeted with small proteins called ubiquitin [102]. Energy consuming degradative process then starts the cascade of events that transfer ubiquitinated substrate to the 26 S proteasome system, and then degradation proceeds. In eukaryotes, compartmentalization makes some organelles more prone to the aggregation of proteins. Proteins in the ER are more prone to errors of glycosylation and disulphide bond formation, so ER and cytosolic quality control for protein differ in dealing with protein damage. Malfunctioning protein quality control mechanisms allow proteins to aggregate and deposit in cells as soluble oligomers and monomers. Later these oligomers can form amyloid fibrils [103]. The transport system in the cell transports aggregates in the cell through microtubular tracks. Amyloid structures are tubular structures generated by globular beta sheet-rich oligomer structures. The inherited pathogenicity in oligomers has been reported widely. However, large aggregates can be eliminated by the autophagy pathway and are cleared from the cell. Polypeptides with exposed hydrophobic stretches are prone to aggregation and play an important role in aggregation assembly [104]. Most neurodegenerative disorders, as listed in Table 1, are associated with aggregated protein deposits. Generally, extracellular amyloid aggregates and intracellular neurofibrillary tangles are the two neuropathological hallmarks of these diseases.

## 4. Approaches to Targeted Protein Aggregation

The strategies are concentrated mainly on the following three points.

1. preventing aggregation by stabilizing the native state
2. refolding of misfolded proteins
3. reinforcing or modifying proteins preventing aggregation, such as post-translational modifications

## 5. Chemical Chaperones for Drug Repurposing in Protein Aggregation Diseases

Cells are exposed to misfolded proteins generated by different processes of life. Inefficient protein biogenesis, mutant proteins, and high concentrations of unstable units of multimeric complexes, also called orphan subunits, are important protein misfolding factors. Inefficient or interruptions in the secretory pathway for protein translocation other than these factors also contribute to protein misfolding and aggregation [105]. Environmental stress, metabolic stress, and diseases such as cancers can enhance the production of misfolded proteins. Elevated temperatures, chemical exposure and biological infections, nutrient imbalance, oxygen stress reactive oxygen species, and mitochondrial deregulations are important factors affecting the homeostatic protein machinery [106]. Environmental stress and cancers have been found to be associated with the overexpression of chaperones [107]. While in the aging process, over time, the protein homeostatic network is overwhelmed by the accumulation of oxidative stress and damage to the proteins leading to the widespread accumulation of protein aggregation products, toxicity, and cell death. To control the damage caused by an accumulation of misfolded and aggregated proteins, the cell has to respond quickly to contain this damage; otherwise, irreversible damage is caused by the toxicity of these structures. Molecular chaperones are central to protein homeostasis maintenance. Cell chaperones not only guide newly synthesized polypeptides to their native structure, but they also help in the translocation of peptides and refolding of denatured intermediates. Chaperones also target misfolded proteins towards proteasome machinery for degradation. Due to this close involvement with proteins in protein folding, some chaperones are linked to protein synthesis and translation machinery [108,109,110].

Aggregation inhibition and prevention of amyloid formation: It is imperative to study mechanisms by which cells manage protein unfolding and the action of the agents engaged in quality control and clearance pathways. Aggresome and inclusion body formation are being extensively studied. It is also important in relation to stress and disease. Proteome maintenance is a major homeostatic duty in the cell [111]. The cell has bewildering mechanisms and several quality control systems to assist in the prevention and clearance of accumulated and misfolded proteins. Disturbed protein homeostasis results in extensive cell and tissue harm. The first instruction for all quality control systems is the recognition of such misfolded proteins. Molecular chaperones have this function. They work as degradation or folding co-factors to assist proper folding [112]. They direct misfolded proteins toward proper folding, or misfolded proteins are directed to the degradative pathway. Despite elaborate systems, unfolded proteins aggregate in cells. Cells resolubilized protein aggregates by molecular chaperones then misfolded proteins are degraded by the ubiquitin-proteasome machinery. When aggregates are insoluble, mechanisms for their clearance from the cell also exist. Autophagy is an important process in the cell. During this process, accumulated material, such as aggregated or misfolded proteins, is engulfed in a membrane structure. The autophagosome fuses with the lysosome, where low pH and endoproteases chop away protein aggregates, and insoluble aggregates are dissolved. Amyloid-β peptide caused Alzheimer’s disease is a good example to understand this process. The frontal cortex and hippocampus in the brain are affected by this disease by the accumulation and deposition of amyloids. Several mechanisms exist for the clearance of amyloid from the brain. Drainage of interstitial fluid, microglial phagocytosis of amyloids, and transport into circulation are some examples. Amyloid degrading enzymes also help clearance, such as neprilysin, a circulating endopeptidase, and matrix metalloproteinase-9. It is an extracellular matrix-degrading enzyme with diverse activity from embryonic development to bone formation and cancer metastasis [113]. In Alzheimer’s disease, neuroinflammation is also an important element in disease progression. Activated microglial cells are a hallmark of Alzheimer’s disease, secreting proinflammatory cytokines. Amyloid β peptide-binding immunoglobulins are available for immunotherapy of Alzheimer’s disease [114]. Humanized monoclonal IgGs are under clinical trials. Humanization is achieved by substituting human IgG sequences for conserved murine IgG sequences sparing complementarity determining region, sequences necessary for Aβ binding. This reduces infusion reactions, induction of neutralizing antibodies to the IgG in human recipients, and anaphylactic reactions [115].

Microtubule-associated protein tau in neurons is also associated with neurodegenerative disease. The important function of tau is to stabilize the microtubule during formation. Hyperphosphorylation of tau has been linked to pathological tau protein leading to aggregation and formation of insoluble neurofibrillary tangles [116]. Intracellular aggregates are accumulated, and there is a loss of soluble tau for microtubule stabilization; combined, these two causes lead to a decline in neural function.

In the brain, microglial cells are important players in the inflammatory process. Microglia are innate immune cells in the brain widely described as migratory phagocytic cells in the nervous system. They are the most abundant mononuclear phagocytes in the brain and contribute to homeostasis and brain development from very early life stages. Microglia-mediated neuroinflammatory processes are important etiologic events in Alzheimer’s disease. Microglial cells are present in the local area of amyloid plaques and, depending on the expression of a wide range of cytokines, chemokines, and innate immune cell surface receptors, show many activation phenotypes. In progressive disease, microglial cells fail to control amyloid accumulation which causes neurotoxicity of tangles and cellular damage. Inflammation occurs to resolve injury by reducing damage to nearby tissues. In the case of amyloid neurodegenerative diseases, it is intended to clear the brain of damaging forms of Aβ peptides. The appropriate triggering and activation of the microglial system can help in the resolution of cerebral amyloids in animal models. Both transplanted and bone marrow-derived microglia have shown a role in plaque clearance [117]. Microglial cells originate from peripherally derived myeloid lineage progenitor cells. They are brain tissue macrophages and primary immune effector cells. Their copiousness varies considerably between brain areas. For every few hours of rest, microglia are capable of screening the whole brain parenchyma. In case of injury, microglial cells migrate to the lesion sites to identify pathogens and help clean up the site by installing the proper immune response.

Microglia in the brain show phenotypic and functional differences compared to peripheral macrophages. Microglial cells are out-of-the-way from circulation by the blood-brain barrier (BBB) during the course of their development at early embryonic stages. Another kind of myeloid cell termed perivascular macrophages, lines blood vessel walls in the central nervous system. Perivascular macrophages are phenotypically similar to circulating peripheral macrophages. Perivascular macrophages have been found to be very important in clearing vascular amyloid deposits in brain parenchyma together with microglia and infiltrating macrophages [118,119,120]. The amyloid β peptide is a primary constituent of senile plaques in Alzheimer’s disease. This plaque formation is critical for the dysfunction of neurons during AD progression. Amyloid β oligomers are neurotoxic and can raise extrasynaptic glutamate levels, extrasynaptic N-methyl-Daspartic acid receptor-mediated excitotoxicity, and postsynaptic depression. Other effects of amyloid β oligomers include calcium balance, impaired mitochondrial function, and induction of reactive oxygen species. All these developments eventually lead to neuronal apoptosis and cell death.

**Figure 6:**
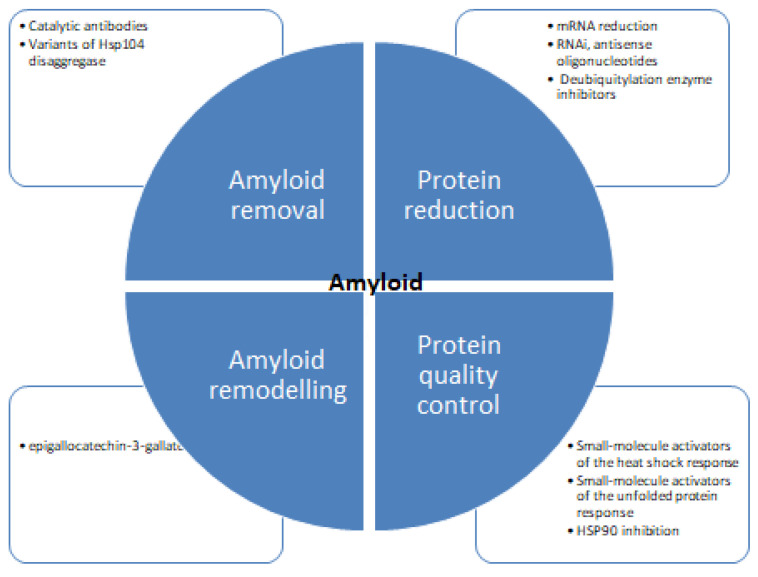
Therapeutic strategies for protein aggregation diseases [75,119,120].

Despite concentrated research, the mechanism of protein inhibition is poorly understood. Figure 6 shows different strategies for tackling protein aggregation. There are several recent studies available detailing the structural aspects of protein folding, and also there are many studies focused on the inhibition of amyloid formation by small molecules. There are diverse groups of molecules proven effective against protein aggregation and fibrillation [121,122]. These molecules impede the process of aggregation and hinder the self-assembly of the polypeptide. Some molecules stabilize the native state of proteins and minimize the possibility of protein aggregation [123,124,125,126]. Several natural polyphenols such as nordihydroguaiaretic acid, epigallocatechin 3-gallate, catechins, flavanols, and stilbenes have been reported to inhibit protein aggregation in vitro [127,128,129]. There are many studies suggesting the antioxidative, anti-inflammatory, and neuroprotective properties of these molecules can prevent amyloid stress in cells. Many in vivo studies showed polyphenols to inhibit amyloid deposits in brain tissue. Such as curcumin, injected into mice, where it peripherally crossed the blood-brain barrier reducing amyloid levels and improving cerebrovascular amyloid angiopathy [130]. Another study with polyphenol-rich grape seed extract diet-fed mice showed a decrease in Aβ deposition in mode animals [131]. Diets rich in gallic acid [132], catechin [133], epicatechin and myricetin [134], nordihydroguaiaretic acid [135], rosmarinic acid [136], and resveratrol [137] also showed diminished plaque formation in different parts of brain and alleviation of symptoms. Polyphenols and structure-based therapeutic molecules can help in the development of therapeutic agents for fighting amyloid diseases. It is imperative to find effective therapies to be identified to treat such diseases. Antisense oligonucleotides represent a novel therapeutic potential to treat protein aggregation and other rare diseases [138,139,140,141]. Recent progress in scientific understanding and methodological advances in the design, synthesis, and targeting of brain mRNA and microRNA with synthetic antisense oligonucleotides has shown promising results. Several types of antisense oligonucleotidesallow the utilization of different mechanisms of posttranscriptional regulation and offer enhanced effects over alternative therapeutics.

## 6. Conclusions

Cellular and molecular mechanisms naturally tend to prevent protein misfolding and aggregation. Chaperones sort the proteins for folding, degradation, and sequestration in inclusion bodies. Although the exact mechanism for this sorting is not well understood but is central in cellular protein quality control. Inclusions of misfolded proteins have been found commonly in bacteria, especially for overexpressed recombinant proteins. Inclusion formation generally starts when other cellular proteasome control mechanisms are overwhelmed. Environmental and metabolic short-term stress also initiate inclusion body formation in cells. Sometimes, this process is reversible. The formation of insoluble amyloids is a hallmark of many neurodegenerative protein aggregation diseases such as Alzheimer’s and Huntington’s disease. Amyloid disease-causing proteins are broadly divided into two classes. Globular proteins and natively unfolded proteins are characterized based on their native structures. There are structural similarities presented in amyloid-associated proteins in various amyloid diseases. Partial unfolding is required for globuar proteins to attain self-associated β-sheetrich toxic aggregates. Examples of such proteins include prion protein, macroglobulin, and transthyretin. Natively unfolded proteins include tau proteins, β amyloid-protein, and α-synuclein. These proteins also undergo folding changes generating unstable intermediate-like structures of globular proteins, which subsequently undergo conversion to β-sheet-rich structure and self-assembly by conformational transformations. The hallmark of amyloid diseases is the deposition of fibrillar proteinaceous material intracellularly or extracellularly. Cellular inclusions are not always insoluble. Recent research shows that these inclusions can serve as a depot of proteins with cellular pool exchange. However, aggregation is not always useful in the cell. There are many approaches to deal with the aggregation problem. There have been reports that drugs can target preformed fibrils. Short peptides such as TNGQ inhibited preformed amyloid fibrils of islet amyloid polypeptide. Levodopa functionalized nanoparticles showed promising results against aggregation of the amyloid beta protein. Conjugated polymers, polyphenols, and macrocyclic compounds are other candidates that have proven aggregation inhibition potentials. However, there is still a long way to cover to reach any consensus for applying these compounds clinically. There are reports that many promising compounds that were experimented on in human subjects showed toxicity levels that were unexpected, and then they were withdrawn. There are many aspects to ponder and many dimensions to explore in this area of research. There is an urgent need for intensive investigation in this area so that we will have some potent, less toxic treatment options for protein conformational disease treatment.

In the present review, we tried to include different aspects pertaining to protein aggregation and how our cells react to these conditions. There are different aspects of protein aggregation. On the one hand, failure to achieve proper protein folding results in aggregation and disruption of cell homeostasis leading to debilitating, degenerative disorders. While, On the other hand, protein structures with similar conformational attributes to those disease-causing proteins are being found to show functional roles elsewhere. The battery of analyses methodology and instrumentations are being used to generate structural and functional information about protein aggregation. Natural selection pressure has polished protein sequences that sometimes fold into stable and ordered functional amyloids. The same force also prevented the accumulation of cytotoxic protein aggregates and assemblies. Untangling the mysterious biased handling of proteins in two cases is not an easy task. The problem is complicated owing to the similarity in the types of interactions in both assemblies and the types of interaction forces operating to attain the native and aggregated state are essentially the same. A possible solution is to focus on aggregation pathways as a replacement for mature structures. This information we can use to generate anti-amyloid treatments

Future direction: Focussed research is required to specifically identify conformational transitions in the early stages of the aggregation process; these sites can serve as important targets for aggregation inhibition. Such studies can boost the improvement of strategies currently in use to counteract the aggregation of proteins at different stages of protein aggregation. It is now imperative to have a synergistic research approach to tackle this issue. The basic information about the mechanism of aggregation has the potential to be translated into novel therapeutics. These are important methods to generate a cure for devastating amyloid disorders.

## Figures and Tables

**Table 1:** Amyloid-associated proteinopathies.

Proteinopathies	Protein Involved
Alzheimer’s Disease	Beta Amyloid
Finnish Amyloidosis	Gelsolin
Medullary Carcinoma of the Thyroid	Calcitonin
Senile Systemic Amyloidosis	Transthyretin
Prolactinomas	Prolactin
Rheumatoid Arthritis	Serum Amyloid A
Huntington’s Disease	Huntingtin
Diabetes Mellitus Type 2	Iapp (Amylin)
Hereditary Non-Neuropathic Systemic Amyloidosis	Lysozyme
Parkinson’s Disease	Alpha-Synuclein
Dialysis Related Amyloidosis	Β2 Microglobulin
Familial Amyloid Polyneuropathy	Transthyretin
